# Polymorphism in *BACH2* gene is a marker of polyglandular autoimmunity

**DOI:** 10.1007/s12020-021-02743-9

**Published:** 2021-05-08

**Authors:** Marta Fichna, Magdalena Żurawek, Bartosz Słomiński, Marta Sumińska, Agata Czarnywojtek, Natalia Rozwadowska, Piotr Fichna, Małgorzata Myśliwiec, Marek Ruchała

**Affiliations:** 1 Department of Endocrinology, Metabolism and Internal Medicine, Poznan University of Medical Sciences, Poznan, Poland; 2 Institute of Human Genetics, Polish Academy of Sciences, Poznan, Poland; 3 Department of Medical Immunology, Faculty of Medicine, Medical University of Gdansk, Gdansk, Poland; 4 Department of Paediatric Diabetes and Obesity, Poznan University of Medical Sciences, Poznan, Poland; 5 Department of Pharmacology, Poznan University of Medical Sciences, Poznan, Poland; 6 Department of Paediatrics, Diabetology and Endocrinology, Faculty of Medicine, Medical University of Gdansk, Gdansk, Poland

**Keywords:** Autoimmune polyglandular syndrome, Autoimmunity, *BACH2*, Multiple organ autoimmunity, Polymorphism

## Abstract

**Purpose:**

Genetically predisposed individuals may develop several autoimmune diseases—autoimmune polyendocrine syndromes (APS). APS types 2–4, are complex disorders, which combine various organ-specific autoimmune conditions. Recent reports support the considerable role of the *BACH2* gene in immune cell differentiation and shifting the T-cell balance towards regulatory T-cells. *BACH2* polymorphisms are associated with autoimmune disorders, including Addison’s disease (AD), Graves’ disease (GD), and probably type 1 diabetes (T1D). Our study was aimed to investigate the *BACH2* variant, rs3757247, in endocrine autoimmunity in the Polish population.

**Methods:**

The analysis comprised 346 individuals with APS, 387 with T1D only, and 568 controls. Genotyping was performed using TaqMan chemistry.

**Results:**

APS type 2 was found in 219 individuals, type 3 in 102, and type 4 in 25 subjects. Overall, AD was diagnosed in 244 subjects, Hashimoto’s thyroiditis—in 238, T1D—in 127, GD—in 58, vitiligo and chronic gastritis each in 40 patients, celiac disease—in 28, premature menopause in 18, and alopecia in 4 patients. Minor T allele at rs3757247 was found in 56.4% APS vs. 44.1% control alleles (OR 1.59; 95%CI: 1.30–1.95, *p* < 0.0001). The distribution of genotypes revealed excess TT homozygotes in the APS cohort (33.2 vs. 20.1% in controls, *p* < 0.0001). The frequencies of rs3757247 alleles and genotypes in T1D patients did not present significant differences vs. controls (*p*-values > 0.05).

**Conclusions:**

These results provide evidence of the association between *BACH2* polymorphism and polyglandular autoimmunity. Since carriers of rs3757247 display increased risk for additional autoimmune conditions, this variant could identify individuals prone to develop APS.

## Introduction

Autoimmunity accounts for up to one hundred diseases, which, taken together, are responsible for considerable morbidity and mortality worldwide [1]. In most instances, the etiology of these disorders remains obscure and combines some poorly determined environmental triggers with complex inherited susceptibility, conferred by numerous genetic polymorphisms [2]. Autoimmune conditions tend to cluster within families, although their inheritance pattern is rarely straightforward [3, 4]. Furthermore, genetically predisposed individuals may develop several autoimmune diseases, traditionally categorized into four distinct autoimmune polyendocrine syndromes (APS) [5]. This canonical classification, established by Neufeld and Blizzard, enables to distinguish APS type 1, which is a unique monogenic entity, APS type 2, which comprises Addison’s disease (AD) combined with autoimmune thyroid disease (AITD) and/or type 1 diabetes (T1D) (and possibly any other autoimmune disease), APS type 3, which designates any combination of autoimmune diseases without Addison’s disease (e.g., AITD together with chronic atrophic gastritis [CAG]), and finally, APS type 4, which includes any combination of AD with autoimmune conditions other than AITD and T1D, such as CAG, or vitiligo [5]. Some authors simply distinguish APS type 1 due to autosomal recessive mutations in the *AIRE* gene, and APS type 2, for all other autoimmune disease combinations that do not follow mendelian inheritance [6].

Subjects suffering from several autoimmune conditions most probably display a general immune defect, predisposing them to multi-target auto-aggression. Scarce published studies on non-type 1 APS revealed that individuals with polyglandular autoimmunity present impaired function of T regulatory (Treg) CD4 + CD25 + cells and decreased T cell expression of caspase-3, which takes part in the activation-induced cell death [7, 8]. Accordingly, genes implicated in the immune function are of particular interest with regard to the development of multiple autoimmune conditions. Most of the genetic proneness seems conferred by the HLA variants, which contribute to both mono- and polyglandular autoimmunity [9–13]. Other APS susceptibility genes include *CTLA4* involved in co-stimulatory signal delivery, *PTPN22* engaged in downstream T cell signaling, and TNF-alpha, a proinflammatory cytokine, that mobilizes the immune cells [14, 15]. Nonetheless, genetic data with regard to polyendocrine autoimmunity remain sparse, and our knowledge is often inferred from monoglandular autoimmune diseases, such as T1D, Hashimoto’s thyroiditis (HT), Graves’ disease (GD), and AD alone.

Recent reports support the considerable role of the *BACH2* gene in immune function and single organ autoimmunity. *BACH2* is located on chromosome 6q15 and encodes one of the two transcription factors belonging to the Bach (BTB domain and Cap‘n’Collar homolog) family, implicated in immune response control through transcriptional repression of the target genes [16]. Initially, its expression seemed restricted to monocytes and neuronal cells, but further studies revealed high *BACH2* levels in B-cell lineage and its critical role in the final differentiation of the mature B cells into antibody-secreting plasma cells [16, 17]. Current evidence shows that Bach2 protein is also expressed in T cells, where it takes part in shifting T cell balance towards the development of Treg cells while limiting the expression of genes required for effector CD4 + T cell differentiation into Th1, Th2, and Th17 lineages [18, 19]. Bach2-deficient mice present Treg cell depletion, diminished Foxp3 expression, and their aberrant differentiation with enhanced Th2 cytokine production, leading to fatal inflammatory lung disease [19, 20]. In humans, *BACH2* haploinsufficiency results in an immunodeficiency-autoimmunity syndrome with lymphocyte maturation defects that cause immunoglobulin deficiency, recurrent broncho-pulmonary infections, and intestinal inflammation [21]. Polymorphisms within the *BACH2* locus were found to be associated with several autoimmune conditions, comprising vitiligo, rheumatoid arthritis, and systemic lupus erythematosus [21–24]. As for the endocrine disorders, *BACH2* variants were associated with adrenal and thyroid autoimmunity [25–28]. Data from T1D cohorts are unequivocal—genome-wide association studies pinpointed *BACH2* polymorphisms as risk factors, while some local studies, including a small Polish series, failed to replicate this association [29–32]. With these in mind, our study was aimed to explore the genetic variant of the *BACH2* gene, rs3757247, in autoimmune endocrine disease among Polish subjects.

## Materials and methods

The analysis was conducted in a cohort of 775 Caucasian patients (349 males, 426 females) suffering from autoimmune endocrine disorders and 568 healthy controls (266 males, 302 females). All patients were enrolled in the tertiary endocrine and diabetes reference centers at Poznan and Gdansk Universities of Medical Sciences. The diagnosis of T1D was based upon WHO glycaemic criteria with an absolute dependence on exogenous insulin. Clinical diagnosis of primary adrenal failure was confirmed by either low basal serum cortisol with concomitant high plasma adrenocorticotropin level, or insufficient cortisol response to the short Synacthen test (250 µg parenteral synthetic ACTH_1-24_) [33]. AITD disease, either HT or GD, was diagnosed with relevant hormonal (thyroid-stimulating hormone, free thyroxine, and free triiodothyronine) and serologic results, accompanied by typical heterogeneous, hypoechoic ultrasound image of the thyroid gland [34]. Autoimmune etiology of the endocrine disorders was corroborated by positive serum autoantibodies to insulin, glutamic acid decarboxylase and/or tyrosine phosphatase in T1D, autoantibodies to 21-hydroxylase in AD, and autoantibodies to thyroid peroxidase (aTPO), thyroglobulin (aTg) in HT, and to TSH receptor in GD [35]. Patients with type 1 APS (AD with mucocutaneous candidiasis, hypoparathyroidism, and/or ectodermal dystrophy) were excluded from the study. Premature ovarian insufficiency (POI) was diagnosed in females if their menopause occurred before the age of 40 years old and was confirmed by elevated serum follicle-stimulating hormone and ovarian ultrasound image. Data with regard to celiac disease (CD) (serum IgA to tissue transglutaminase and histological evaluation of duodenal mucosa according to Marsh classification) and chronic atrophic gastritis with/without the ensuing pernicious anemia (circulating parietal-cell autoantibodies and histological assessment of gastric mucosa by updated Sydney system, with/without decreased serum vitamin B12) were extracted from patients’ preexisting medical records. Vitiligo and alopecia were diagnosed based on clinical examination.

Control subjects were recruited among healthy blood donors with negative history of autoimmunity, no clinical signs of autoimmune disorders, and fasting plasma glucose within the reference range. Moreover, to minimize the risk of the ongoing subclinical HT, which is one of the most common autoimmune diseases, healthy individuals were screened for the presence of circulating aTPO.

Genomic DNA was extracted from the peripheral blood using Gentra Puregene Blood Kit (Qiagen, Hilden, Germany). Genotyping of rs3757247 was performed by real-time PCR using commercial Taqman SNP Genotyping assay (C_27475051_10) on CFX96 Real-Time Detection System (BioRad Laboratories, CA, USA) and following the conditions recommended by the manufacturer (Applied Biosystems by Thermo Fisher Scientific, USA). Genotypes were confirmed by direct DNA sequencing of both strands by BigDye Terminator Cycle Sequencing Ready Reaction Kit on ABI Prism 3730 Genetic Analyzer (Foster City, CA, USA). To ensure the genotyping accuracy 7% of samples were genotyped in duplicate.

The power estimation, performed with PS Power and Sample Size calculator v.2.1.30 (Vanderbilt University, TN) assuming an allelic odds ratio (OR) of 1.4, similar to that reported in the former UK/Norwegian study, and given the minor allele frequencies as observed in the control group, showed 93.4 and 92.4% power to detect effects in our APS and T1D cohorts, respectively (*α* = 0.05) [28]. Hardy–Weinberg equilibrium of rs3757247 genotypes was checked in all three cohorts (threshold *p* > 0.05) using a Chi-squared test in an online calculator available at the Helmholtz Center Munich website (http://ihg.gsf.de/cgi-bin/hw/hwa1.pl). Chi-squared test was also applied for statistical association analysis on 2 × 2 and 2 × 3 contingency tables. Statistical calculations were performed using GraphPad Prism 6.0c (GraphPad Software, La Jolla, CA).

## Results

Clinical analysis of the patients’ cohort enabled us to distinguish a group of 346 individuals with APS and a series of 387 subjects suffering solely from T1D. In line with disease characteristics, the number of patients with isolated form of AD was limited, comprising just 42 individuals, which precluded further reliable analysis of *BACH2* polymorphism in this subgroup. The mean age of patients with APS was 39.4 ± 17.9 years, and those with T1D only was 24.3 ± 10.4 years. The APS cohort comprised 219 individuals with APS type 2, 102 with APS type 3, and 25 with APS type 4. AD was diagnosed in 244 subjects, HT—in 238 participants, T1D—in 127 individuals, GD—in 58, vitiligo and CAG each in 40 patients, CD—in 28 subjects, POI in 18 females, and alopecia in four patients. The most common disease combination was AD with HT, which affected 101 individuals, and 58 further subjects with AD, HT, and additional autoimmune condition(s) (Table 1). Another common option was HT coexisting with T1D, found in 57 subjects, and in 36 more individuals who, apart from HT and T1D, also displayed other coexisting autoimmune diseases. In the studied cohort of 346 patients with polyglandular autoimmunity, 255 (73.7%) subjects were diagnosed with just two disorders, 77 (22.3%)—with 3 autoimmune diseases, and 14 (4.0%)—with 4 autoimmune conditions.

**Table 1:** Most frequent (at least 5 cases) combinations of the autoimmune diseases in the studied cohort of 346 patients with polyglandular autoimmunity

Disease combination	Number of patients	%
AD + HT	101	29.2
HT + T1D	57	16.5
AD + GD	38	11.0
AD + HT + V	14	4.0
AD + HT + CAG	14	4.0
T1D + CD	14	4.0
AD + HT + T1D	12	3.5
AD + CAG	10	2.9
GD + T1D	10	2.9
AD + HT + POI	9	2.6
AD + HT + T1D + V/CD/POI/CAG	9	2.6
HT + T1D + CD	8	2.3
HT + T1D + V/CAG/POI	7	2.0
AD + T1D	5	1.4
AD + T1D + GD/V/CD	5	1.4

*AD* Addison’s disease, *CAG* chronic atrophic gastritis, *CD* celiac disease, *HT* Hashimoto’s thyroiditis, *GD* Graves’ disease, *POI* premature ovarian insufficiency, *T1D* type 1 diabetes, *V* vitiligo

Genotype frequencies of the studied *BACH2* polymorphism remained in Hardy–Weinberg equilibrium in all considered cohorts (*p* = 0.246 for APS cohort, *p* = 0.314 for T1D group, and *p* = 0.549 for controls). The T allele at rs3757247 was found in 390 of 692 (56.4%) APS patient alleles compared to 501 of 1136 (44.1%) healthy control alleles, yielding an OR of 1.59 (95% CI: 1.30–1.95, *p* < 0.0001). The difference in the distribution of rs3757247 genotypes also revealed statistical significance, with considerable excess of TT homozygotes in the APS cohort (33.2 vs. 20.1% in controls, *p* < 0.0001). On the contrary, the frequencies of rs3757247 alleles and genotypes did not present significant differences between patients suffering from T1D and controls (*p*-values 0.501 and 0.839, respectively). Full allele and genotype data are displayed in Table 2.

**Table 2:** Genotype and allele frequencies of rs3757247 *BACH2* variant in patients suffering from autoimmune polyglandular syndrome (APS), type 1 diabetes (T1D), and healthy controls (CON)

rs3757247	APS (%)	T1D (%)	CON (%)
Genotypes			
CC	71 (20.6)	114 (29.5)	181 (31.9)
CT	160 (46.2)	201 (51.9)	273 (48.0)
TT	115 (33.2)	72 (18.6)	114 (20.1)
*p*-value	<0.0001	0.501	
Alleles			
C	302 (43.6)	429 (55.4)	635 (55.9)
T	390 (56.4)	345 (44.6)	501 (44.1)
*p*-value	<0.0001	0.839	

Further stratification of the APS cohort by the number of coexisting autoimmune disorders did not reveal significant differences in rs3757247 distribution between patients affected with just two and those suffering from three and more autoimmune conditions (*p* = 0.214 for genotype-wise, and *p* = 0.142 for allele-wise analysis) (data not shown).

## Discussion

The results of the current study provide evidence of the association between *BACH2* polymorphic variant and autoimmune polyglandular disease. Homozygous carriers of the minor rs3757247 allele appeared at particularly increased risk for multiple autoimmune conditions, although no effect on a number of disorders was detected. For the purpose of this study, we did not differentiate between APS types 2, 3, and 4. Nowadays, most authors simply distinguish monogenic (type 1) and polygenic (type 2–4) APS, also referred to as juvenile and adult APS, respectively [6, 36]. Still, considering their genetic background, specifically HLA alleles, some researchers advocate for the traditional further subdivision of type 2 APS [13]. However, prospective observations of patients with APS indicate that new autoimmune disorders may emerge at any age, shifting individual classification from APS type 3 to type 2 or 4. This is especially true for females, in whom mean age at AD onset is close to 40 years [37]. Our eldest patient, already suffering from HT and vitiligo for several years, was diagnosed with autoimmune adrenal failure at the age of 71 years. Furthermore, as illustrated in Table 1, even though only nine autoimmune conditions were taken into account, several disease combinations could be observed. In our cohort, AITD was the most common autoimmune disease, found in nearly 300 individuals (238 cases of HT and 58 of GD), while the number of AD cases (244 individuals) closely followed thyroid autoimmunity. AITD, and HT in particular, are quite common diseases, reaching up to 5% of the general population [38]. However, only a small proportion of subjects with AITD actually develops additional autoimmune disorders, whereas concomitant diseases are found in more than 50% of patients with AD [37, 39, 40].

Our experience with patients suffering from autoimmune endocrine disorders demonstrates that this group requires careful periodic evaluation towards new entities which may develop. It allows the timely introduction of the replacement therapy, decreases acute morbidity and mortality, and potentially enables longer preservation of the affected gland function. Although many autoimmune diseases do not cause serious complications, some constellations, such as AD adding up to T1D, or GD overlying pre-existing AD may be dangerous, even life-threatening, leading to episodes of severe hypoglycemia or adrenal crisis, respectively [41, 42]. Tools enabling prior identification of subjects at particular risk of multiple autoimmunities could indicate who might benefit from regular serologic and biochemical analyses to prevent devastating complications. Population screening is definitely not cost-effective in this case but could be reasonably restricted to individuals at risk, such as patients with pre-existing autoimmune disease, which may exacerbate with the advent of an additional autoimmune disorder, and relatives of subjects with non-type 1 APS, who seem particularly susceptible for autoimmunity, but in some families do not develop autoimmune disorders at all. Well-designed, validated genetic screening panel could potentially further refine the autoimmune risk estimation, and if autoimmunity was deemed not likely, relieve many subjects of the burden of repeated serologic and biochemical investigations [43]. Molecular makeup, including single nucleotide polymorphisms, remains stable over a lifetime, so there is no need to evaluate it several times [44]. On the contrary, serum autoantibodies, although invaluable markers, may appear at any time, hence even if absent at the moment, there is no guarantee that they will not emerge at some point in the future. Moreover, with decreasing costs of the high-throughput molecular techniques, prices of genotyping screens are becoming widely available. For these reasons, polymorphic variants of the genes associated with increased susceptibility for autoimmune conditions seem logical candidates for a screening panel.

Major autoimmune endocrine disorders share much of their molecular background, including the strongest genetic factor, i.e., class II HLA haplotypes, DR3/DQ2, and DR4/DQ8 [9–12, 45]. Polymorphisms in two other genes, *CTLA4* and *PTPN22*, both implicated in lymphocyte function, are associated with T1D, AITD, and AD [46–49]. Recent data confirm that carriers of the risk alleles *PTPN22* 1858T and *CTLA4* CT60 are more prone to develop several autoimmune conditions [15]. By analogy, it seemed plausible that *BACH2* gene polymorphism, implicated in lymphocyte differentiation and function, might equally promote multitarget autoimmunity. It demonstrated association with several autoimmune conditions, systemic as well as organ-specific [21–30]. In the context of endocrine autoimmunity, the *BACH2* locus was first identified by means of a meta-analysis of the first genome-wide association studies in T1D [29, 30]. Soon afterward it was also pinpointed (rs11755527) with regard to thyroid autoimmunity—it displayed an association with GD, and also with circulating aTPO in patients suffering from T1D [25]. These findings were further confirmed in British, Chinese Han, and multinational Caucasian populations [26, 50, 51]. The association between *BACH2* rs11755527 and T1D was then replicated among Pakistani patients but failed to be confirmed in Brazilians [32, 52]. A former investigation of rs3757247 in adult T1D patients (mean age 33 ± 10 years) from Western Poland also revealed negative results, however, the studied cohort was limited to just 141 individuals hence that analysis could be underpowered to detect a minor effect [31]. Furthermore, experimental data demonstrated that *BACH2* is expressed in pancreatic islets, where it may be involved in the regulation of cytokine-induced beta-cell apoptosis via activation of the JNK1/BIM pathway [53]. These observations have prompted us to verify the association between *BACH2* polymorphism and T1D in Polish patients. However, despite a much larger sample size, we were not able to discern an effect. Both *BACH2* variants evaluated in various studies, rs3757247 and rs11755527, are located in intron 3 and remain in tight linkage disequilibrium (*r*^2^ value 0.92) therefore this could not be the reason for discrepant results [28]. Most probably, population differences are responsible, or alternatively, the effect size of *BACH2* with regard to T1D alone is indeed very modest.

Nonetheless, our study clearly demonstrates an association of rs3757247 with polyglandular autoimmunity, in a large cohort comprising individuals with APS type 2, 3, and 4. The Swedish authors were the first to recognize that a high rate of comorbidities makes AD a good natural model to study the shared genetic basis for autoimmunity. Their analysis based upon exome sequencing of nearly 2000 immune-related genes pinpointed *BACH2* as a risk locus in AD (rs62408233), which remained statistically significant even after recalculating the logistic regression concerning only the cases with isolated AD and without circulating aTPO [27]. *BACH2* variant, rs3757247 was independently explored in another study, comprising the UK and Norwegian AD cohorts, which confirmed increased frequency of TT genotype among the affected subjects [28]. This association was detectable in AD patients suffering from type 2 APS (both in 195 UK and in 167 Norwegian subjects) and those with isolated AD (in 165 UK patients) as well [28]. In accordance, former analysis in a large series of T1D patients tested for several organ-specific autoantibodies revealed an association between rs11755527 and serum antibodies to tyrosine phosphatase IA-2, to thyroid peroxidase, and a borderline relationship with circulating antibodies to the major adrenocortical antigen, 21-hydroxylase [54]. These results further support *BACH2* involvement in multiple target autoimmunity. As an intronic *BACH2* polymorphism, rs3757247 is rather unlikely to alter the gene function, however, it may remain in linkage with a genuine causative variant located somewhere else within the locus [28]. Nonetheless, the results of our study, conducted in an ethnically homogenous, clinically well-characterized APS cohort, indicate that rs3757247 is apparently one of the universal genetic markers of autoimmunity, such as HLA, *PTPN22*, *CTLA4*. However, establishing a reliable set of polymorphisms to predict multiplex autoimmunity, is a matter of further cross-sectional association analyses in several populations, followed by prospective validation studies in at-risk cohorts. Since the ongoing progress in immunomodulatory therapies may one day allow targeted preventive interventions in autoimmune endocrine disease, reliable identification of subjects at high risk of autoimmunity will be cardinal to select appropriate candidates for new treatments [55, 56].

In conclusion, *BACH2* polymorphism confers susceptibility to polygenic autoimmune polyglandular syndromes. Since carriers of rs3757247 display increased risk for additional autoimmune conditions, this variant could be a part of the early diagnostic panel identifying individuals prone to develop multiplex autoimmune endocrine conditions among those diagnosed with a single autoimmune disorder, or in first-degree relatives of patients with APS. Further studies in additional APS cohorts are needed to confirm the predictive clinical utility of our finding.

## Data Availability

The datasets generated during the current study are available from the corresponding author upon reasonable request.
